# The reporting quality of studies investigating the diagnostic accuracy of anti-CCP antibody in rheumatoid arthritis and its impact on diagnostic estimates

**DOI:** 10.1186/1471-2474-13-113

**Published:** 2012-06-25

**Authors:** Elias Zintzaras, Afroditi A Papathanasiou, Dimitrios C Ziogas, Michael Voulgarelis

**Affiliations:** 1 Department of Biomathematics, University of Thessaly School of Medicine, 2 Panepistimiou Str, Larissa, 41110, Greece; 2 The Institute for Clinical Research and Health Policy Studies, Tufts-New England Medical Center, Tufts University School of Medicine, Boston, USA; 3 Department of Gastroenterology, Beth Israel Deaconess Medical Center, Harvard Medical School, Boston, MA, USA; 4 Department of Pathophysiology, National University of Athens School of Medicine, Athens, Greece

**Keywords:** Rheumatoid arthritis, Anti-cyclic citrullinated peptide 2, Anti-CCP2, Quality, Sensitivity, Specificity, Meta-analysis

## Abstract

**Background:**

Recently anti-CCP testing has become popular in the diagnosis of rheumatoid arthritis (RA). However, the inadequate reporting of the relevant diagnostic studies may overestimate and bias the results, directing scientists into making false decisions. The aim of the present study was to evaluate the reporting quality of studies used anti-CCP2 for the diagnosis of RA and to explore the impact of reporting quality on pooled estimates of diagnostic measures.

**Methods:**

PubMed was searched for clinical studies investigated the diagnostic accuracy of anti-CCP. The studies were evaluated for their reporting quality according to STARD statement. The overall reporting quality and the differences between high and low quality studies were explored. The effect of reporting quality on pooled estimates of diagnostic accuracy was also examined.

**Results:**

The overall reporting quality was relatively good but there are some essential methodological aspects of the studies that are seldom reported making the assessment of study validity difficult. Comparing the quality of reporting in high versus low quality articles, significant differences were seen in a relatively large number of methodological items. Overall, the STARD score (high/low) has no effect on the pooled sensitivities and specificities. However, the reporting of specific STARD items (e.g. reporting sufficiently the methods used in calculating the measures of diagnostic accuracy and reporting of demographic and clinical characteristics/features of the study population) has an effect on sensitivity and specificity.

**Conclusions:**

The reporting quality of the diagnostic studies needs further improvement since the study quality may bias the estimates of diagnostic accuracy.

## Background

Rheumatoid arthritis (RA) is a chronic, systemic inflammatory disorder that affects many tissues and organs, mainly synovial joints [1]. The disease leads progressively to the destruction of articular cartilage and ankylosis of the joints [2]. Although the cause of RA is unknown, autoimmunity plays a pivotal role in both its chronicity and progression [3]. RA affects females more frequently than males and it is diagnosed mainly in age 40–60 years [4].

The diagnosis of RA is based on clinical criteria and laboratory tests. Regarding the later tests, the presence of the rheumatoid factor (RF), an autoantibody, consists one of the American College of Rheumatology (ACR) criteria for presence and severity of RA [5]. However, RF has a limited specificity since it can be detected in other autoimmune or infectious diseases, and in the healthy elderly. Anti-cyclic citrullinated protein antibodies (anti-CCP) are other autoantibodies that may be detected in RA patients. Recently anti-CCP testing has become substantial part of ACR-EULAR classification criteria for RA [6]. There is evidence that CCP-assays provide comparable performance with that of RF [7]. However, analysis of the association between anti-CCP antibody titre and RA activity produced contradictory results [8,9]. Anti-CCP2 assay is the most popular because of its high diagnostic specificity and its predictive and prognostic value in RA [10-12].

Currently, diagnostic studies on anti-CCP assays are publishing with a high rate [13]. However, overestimated and biased results from poorly designed and reported studies may direct scientists into making false decisions [14-16]. The reporting information on design and conduct of diagnostic studies is crucial, though, its absence has already been noticed [17,18]. Nevertheless, appropriate reporting may allow researchers to detect potential bias in studies’ internal validity, to assess generalizability and applicability of their results [19]. A survey of published studies of diagnostic accuracy showed that the methodological quality was not optimal. In addition, information on issues like study design, conduct and data analysis was often not reported [20,21].

Inadequate reporting of the published diagnostic accuracy studies may restrict the generalizability, applicability and credibility of studies’ results. A number of guidelines and statements have been developed to improve the quality of a variety of study designs [22], including the diagnostic accuracy studies [19]. In particular, in order to improve the reporting of diagnostic accuracy studies, the Standards for Reporting of Diagnostic Accuracy (STARD) statement has been proposed (http://www.stard-statement.org/)[19]. The STARD statement is a checklist of 25 criteria that diagnostic accuracy studies should conform to in order to make their conclusions easier to assess, interpret and generalize, and lead as a result to better decisions in diagnosis. However, STARD does not assess the actual quality of the research study but the reporting quality, two issues which are not necessarily correlated. In addition to STARD, another tooled has been proposed, called QUADAS, for assessing the methodological quality of diagnostic accuracy studies [23]. Recently, QUADAS was used to evaluate the quality of anti-CCP RA studies in a meta-analysis [13].

The aim of the present study was twofold: first, to evaluate the reporting quality of studies used anti-CCP2 for the diagnosis of RA, according to the STARD statement, and second, to investigate whether quality of reporting is associated with the effect size of diagnostic metrics using meta-analytic techniques (data synthesis). The analysis was focused on the reporting of methods and results sections of the STARD statement. The effect of quality on diagnostic accuracy was focused on studies scored as “high quality” and “low quality”, and for specific items of STARD.

## Methods

### Study identification

PubMed was searched for clinical studies, published from January 1987 (date of imposing the revised ACR criteria [5] to September 2010 that assessed the utility of anti-CCP2 assay in the diagnosis of RA. The search used the following strategy: (("diagnosis" or "diagnostic" or "sensitivity" or "specificity") and ("rheumatoid arthritis" or "RA") and ("anti-cyclic citrullinated peptide antibodies" or "anti-CCP" or "antiCCP" or "anti-CCP2" or "antiCCP2")).

The authors independently reviewed the abstracts to determine the eligibility of each article to potentially meet the search strategy. The references of the retrieved articles were also searched. Only articles in English language, published as full papers or short reports were considered in our study. Reviews, editorials, letters and comments were excluded. The agreement level was reported using Kappa statistics.

### Study selection

We included studies that evaluated the utility of anti-CCP2 antibody for diagnosis of RA with more than 10 participants enrolled that provided data sufficient to estimate both sensitivity and specificity. As controls were defined participants free of RA (i.e. diseased with other conditions or healthy). Disagreements were resolved by discussing the full articles.

### Data abstraction

The data were abstracted from each study by two authors (AP and DZ) independently. Data were extracted by using a standardized form that included study setting and technical details of the assay, demographic characteristics of the patients and 2×2 contingency tables (disease status and test outcome) needed to calculate at least the sensitivity and specificity.

When articles reported more than one set of 2×2 data (such as assays data from different manufacturers and/or different cut-offs), then each data set was considered as a different study. Also, articles reported data separately for multiple control groups (diseased, healthy) were considered as separate studies. In overlapping studies, the most recent and/or the largest study was recorded. The agreement level was also reported using Kappa statistics.

### Study quality assessment with STARD

Although all items in the STARD statement are considered important to help to improve the quality of reporting diagnostic accuracy studies, some are more subjective than others to assess potential biases. Thus, in the present study we focused on methodological related items, i.e. the items that correspond to methods and results sections (eleven items in each category). Thus, in total, 22 items were considered (Table 1). In order to determine better if an item is accurately reported in the articles, we took into account the guidance provided by the STARD Explanation and Elaboration document [21]. All items were investigated in terms of whether they were reported, not whether they were actually carried out during the study. Items were to be scored as “yes” if they were reported in enough detail to allow the reader to judge that the definition had been met. Especially in case of item (14) providing participant’s information about the patient recruitment, the item was coded as “yes” only when the flow diagram was given or explicitly described (i.e. the number of controls per case was specified and the matching variables were clearly stated). Alternatives responses (apart from “yes” or “no”) and unclear responses to each item were coded as negative responses. 

**Table 1:** Proportion of reporting of the items in the STARD statement, overall and in a total of 103 diagnostic studies involving rheumatoid arthritis by STARD score group

STARD^*^items	**Overall % of reporting item n = 103**	**% of reporting item**		**P-value‡**
		**Lower quality articles (score < 9)n = 50**	**Higher quality articles (score ≥ 9)n = 53**	
**METHODS**				
**1. The study population: The inclusion and exclusion criteria, setting and locations where the data were collected.**	88.3	78.0	98.1	0.002
**2. Participant recruitment: Was recruitment based on presenting symptoms, results from previous tests, or the fact that the participants had received the index tests or the reference standard?**	98.1	98.0	98.1	0.999
**3. Participant sampling: Was the study population a consecutive series of participants defined by the selection criteria in item 3 and 4? If not, specify how participants were further selected.**	89.3	88.0	90.6	0.756
**4. Data collection: Was data collection planned before the index test and reference standard were performed (prospective study) or after (retrospective study)?**	95.1	90.0	100.0	0.024
**5. The reference standard and its rationale.**	74.8	52.0	96.2	<0.01
**6. Technical specifications of material and methods involved including how and when measurements were taken, and/or cite references for index tests and reference standard.**	57.3	48.0	66.0	0.075
**7. Definition of and rationale for the units, cut-offs and/or categories of the results of the index tests and the reference standard.**	77.7	68.0	86.8	0.032
**8. The number, training and expertise of the persons executing and reading the index tests and the reference standard.**	20.4	8.0	32.1	0.003
**9. Whether or not the readers of the index tests and reference standard were blind (masked) to the results of the other test and describe any other clinical information available to the readers.**	18.4	12.0	24.5	0.130
**10. Methods for calculating or comparing measures of diagnostic accuracy, and the statistical methods used to quantify uncertainty (e.g. 95% confidence intervals).**	90.3	82.0	98.1	0.007
**11. Methods for calculating test reproducibility, if done.**^**#**^	88.0	100.0	86.4	0.999
**RESULTS**				
**12. When study was done, including beginning and ending dates of recruitment.**	41.7	26.0	56.6	0.003
**13. Clinical and demographic characteristics of the study population (e.g. age, sex, spectrum of presenting symptoms, comorbidity, current treatments, recruitment centers).**	87.4	78.0	96.2	0.007
**14. The number of participants satisfying the criteria for inclusion that did or did not undergo the index tests and/or the reference standard; describe why participants failed to receive either test (a flow diagram is strongly recommended).**	12.6	2.0	22.6	0.002
**15. Time interval from the index tests to the reference standard, and any treatment administered between.**	27.2	12.0	41.5	0.001
**16. Distribution of severity of disease (define criteria) in those with the target condition; other diagnoses in participants without the target condition.**	53.4	46.0	60.4	0.169
**17. A cross tabulation of the results of the index tests (including indeterminate and missing results) by the results of the reference standard; for continuous results, the distribution of the test results by the results of the reference standard.**	94.2	92.0	96.2	0.428
**18. Any adverse events from performing the index tests or the reference standard.**	11.7	10.0	13.2	0.761
**19. Estimates of diagnostic accuracy and measures of statistical uncertainty (e.g. 95% confidence intervals).**	74.8	54.0	94.3	<0.01
**20. How indeterminate results, missing responses and outliers of the index tests were handled.**	7.8	6.0	9.4	0.716
**21. Estimates of variability of diagnostic accuracy between subgroups of participants, readers or centers, if done.**^**#**^	100.0	100.0	100.0	-
**22. Estimates of test reproducibility, typically imprecision (as CV) at 2 or 3 concentrations, if done.**^**#**^	96.0	100.0	95.5	0.999

(Lower quality articles, score < 9 and higher quality articles, score ≥ 9).

**#** for smaller number of articles (n = 25 articles for items 11 & 22), (n = 23 articles for item 21).

**‡***P* values were obtained from Fisher’s exact test in order to express the association between proportions for reporting an item across the two groups of articles.

^*^ STARD = Standards for Reporting of Diagnostic Accuracy.

### Estimation of diagnostic accuracy

The estimation of the diagnostic accuracy was based on the sensitivity (Se) and specificity (Sp). Se and Sp were calculated from contingency tables abstracted from each study.

### Data synthesis and analysis

For each study the diagnostic metrics (Se, Sp, positive and negative likelihood ratio) were calculated. A bivariate model [24,25] was used to estimate summary sensitivity and specificity, with 95% confidence and prediction regions around the summary points. Hierarchical SROC analysis that allows for between-study heterogeneity was also applied to four or more studies [25]. Heterogeneity was evaluated visually by using the SROC curve and numerically by using the variance of the logit-transformed sensitivity and specificity. A smaller value of variance indicates low between study heterogeneity. The statistical analysis was performed using Stata v.10 (metandi and metandiplot commands [26]) (StataCorp, College Station, Texas) and SPSS, version 13.0 (SPSS Inc., Chicago).

### Effect of study quality

In addition, to the overall percentages of reporting the STARD statement items, the quality of reporting in high versus low quality articles was explored. Studies were classified as high quality of reporting when quality score ≥ 9 and as lower quality when quality score < 9. The choice of quality score = 9 as cut-off was the median of the overall quality scores of studies. The overall quality score for each article was calculated by summing the weighted score of reported items. A unit weight was applied for each of the item 2, 5, 7, 10, 13, 16 and 19 (considered subjectively more “important”), whereas, a weight of 0.5 for each of the other items. The effect of study quality on diagnostic accuracy was evaluated based on the level of quality (high/low) and on the reporting results of the above “important” STARD items. Then, the estimates of pooled sensitivities and specificities were compared with a z-score test.

## Results

### Eligible studies

**Figure 1:**
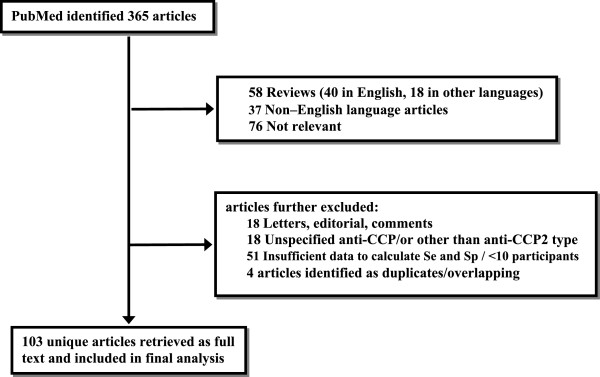
Flow diagram of citations through the retrieval and the screening process.

The literature review identified 364 articles that met the search criteria in PubMed. Thereafter, these articles were retrieved and screened for eligibility. Overall, a total of 103 unique articles remained for analysis having complete full-text evaluation. Figure 1 presents a flow diagram of retrieved articles and articles excluded with specification of reasons. The agreement in article evaluation for eligibility and in extracting the data was both relatively high (kappa = 0.74 (0.70-0.78) and kappa = 0.86 (0.82-0.90)), respectively. A full list of the 103 articles that were retrieved as full-text and included in final analysis is located at the Web site http://biomath.med.uth.gr.

### Study characteristics

The characteristics of studies included in the analysis are shown in Additional file 1: Table S1. A list of journals that endorsed the STARD statement is shown in Additional file 2: Table S2. The 103 eligible articles were published during the period 2003–2010. Consequently, all the eligible articles were published after the introduction of the STARD statement (i.e. 2003). In total 35 different populations (countries) were referred in the eligible articles. Most of the articles conducted in Europe (51 articles, 49.5%) and thereafter in Asia (31 articles, 30.1%), in Africa (7 articles, 6.8%), in North America (7 articles, 6.8%), in South America (6 articles, 5.8%) and in Oceania (1 article, 1.0%). Most of the articles referred to studies conducted in teaching hospitals (52 articles, 50.5%) and the second most frequent studies’ setting was the rheumatologic clinics (31 articles, 30.1%). In 13 out of 103 articles, the detection of anti-CCP2 antibody was done with more than one assay. The four most popular manufacturer assays used, were the Euro-Diagnostica (33 studies, 25.0%), the Axis-Shield (32 studies, 24.2%), the Inova Diagnostics (25 studies, 18.9%) and the Euroimmun (19 studies, 14.4%). A variety of cut-offs were used to define a positive test result according to different manufacturers, but in 9 articles/studies the threshold used was not explicitly given. Control group consisted of participants free of RA (i.e. diseased with other conditions or healthy). From all the above reasons, the 103 articles we had, concluded to a total of 132 studies for the meta-analysis. The mean age of RA participants in the studies, where reported, ranged from 30 years to 70 years (missing information in 37 articles, 35.9%) and the proportion of women RA participants, where reported, ranged from 23.2% to 100% (missing information in 29 articles, 28.2%). Fifty three articles (51.5%) were published in high quality articles (STARD score ≥ 9) and 50 articles (48.5%) in lower quality articles (STARD score < 9) (Table 2).

**Table 2:** Results of Meta-analysis

**Group**	**Articles, n**	**Studies, n**	**Sensitivity (95% ci),%**	**Specificity (95% ci),%**
**All data**	**103**	**132**	**70.8 (68.3-73.2)**	**95.8 (94.9-96.6)**
**Effect of STARD score**				
**High quality score**	53	73	70.9 (67.5-74.1)	95.7 (94.4-96.7)
**Low quality score**	50	59	70.7 (67.0-74.1)	96.0 (94.8-97.0)
**Effect of STARD item 2**				
**Yes**	101	129	70.8 (68.3-73.2)	95.8 (94.9-96.6)
**No**^**¥**^	2	3	NA	NA
**Effect of STARD item 5**				
**Yes**	77	99	71.8 (69.0-74.5)	95.6 (94.5-96.5)
**No**	26	33	67.7 (62.6-72.4)	96.3 (94.9-97.4)
**Effect of STARD item 7**				
**Yes**	80	103	71.0 (68.2-73.7)	95.9 (94.9-96.7)
**No**	23	29	70.0 (64.7-74.9)	95.6 (93.2-97.2)
**Effect of STARD item 10**				
**Yes**	93	121	70.2 (67.5-72.7)*	95.9 (95.0-96.7)
**No**	10	11	77.5 (71.7-82.3)	94.5 (91.7-96.4)
**Effect of STARD item 13**				
**Yes**	90	116	71.5 (68.9-74.1)**	95.6 (94.6-96.4)*
**No**	13	16	65.7 (59.7-71.2)	97.2 (96.2-97.9)
**Effect of STARD item 16**				
**Yes**	55	66	73.3 (69.8-76.6)	94.6 (93.2-95.8)*
**No**	48	66	68.3 (64.9-71.5)	96.8 (95.7-97.7)
**Effect of STARD item 19**				
**Yes**	77	105	70.5 (67.8-73.1)	95.8 (94.8-96.5)
**No**	26	27	72.3 (66.1-77.8)	96.4 (93.8-97.9)

¥ = non-applicable, <4 studies; *P < 0.05, **P = 0.06.

## Main results

Table 1 shows the overall proportion of reporting of the 22 items in the methods and results sections of the STARD statement and the corresponding proportions for high and low quality articles.

Overall, 10 items (six and four items in methods and results sections, respectively) were reported by 85% or more of the studies (Table 1). In methods, the items include the reporting of 1) study population (inclusion/exclusion criteria, setting, location), 2) participants recruitment (eg. based on symptoms, previous testing), 3) participant sampling, 4) data collection (prospective or retrospective study), 5) methods for calculating or comparing measures of diagnostic accuracy and statistical methods used to quantify uncertainty and 6) methods for calculating reproducibility, if done. In results, the items include the reporting of 1) clinical and demographic characteristics of the study population (age, sex, presenting symptoms, comorbidity, current treatment), 2) the cross tabulation or the distribution of the test results by the results of the reference standard, 3) estimates of variability of diagnostic accuracy between subgroups of participants, centers, if done and 4) estimates of test reproducibility, if done.

Furthermore, 13 items (including the ten items already mentioned above) were reported by 70% or more of the studies. The 3 additional items were the reporting of 1) reference standard and its rationale of, 2) definition of and rationale for the units, cut-offs and/or categories of tests results and 3) estimates of diagnostic accuracy and measures of statistical uncertainty.

In contrast, some items were reported only by a small fraction of articles. For example, 20% of articles provided the number, training and expertise of persons executing the tests, 18% reported the blinding status, 13% provided information on recruitment, 12% reported adverse events and finally, 8% provided details about handling of missing responses and outliers.

### Effect of study quality

In comparing the quality of reporting in high quality (quality score ≥ 9) versus lower quality (quality score < 9) articles, significant differences were seen in 11 items (P < 0.05) (6 items in methods: study population, data collection, reference standard, definition of units/cut-offs, number/training/expertise of persons executing the tests, methods for calculating diagnostic measures and 5 in results: dates of recruitment, clinical/demographic characteristics, information on recruitment, time interval between tests, estimates of diagnostic accuracy). In all these items high quality articles showed better performance. An item-by-item comparison is presented in Table 1.

### Impact of study quality on diagnostic estimates

Table 2 shows the meta-analysis’ overall results (pooled sensitivities and specificities), the results according to STARD score (high/low quality) and the results for specific STARD items (comparison of outcome “yes” vs. “no”).

**Figure 2:**
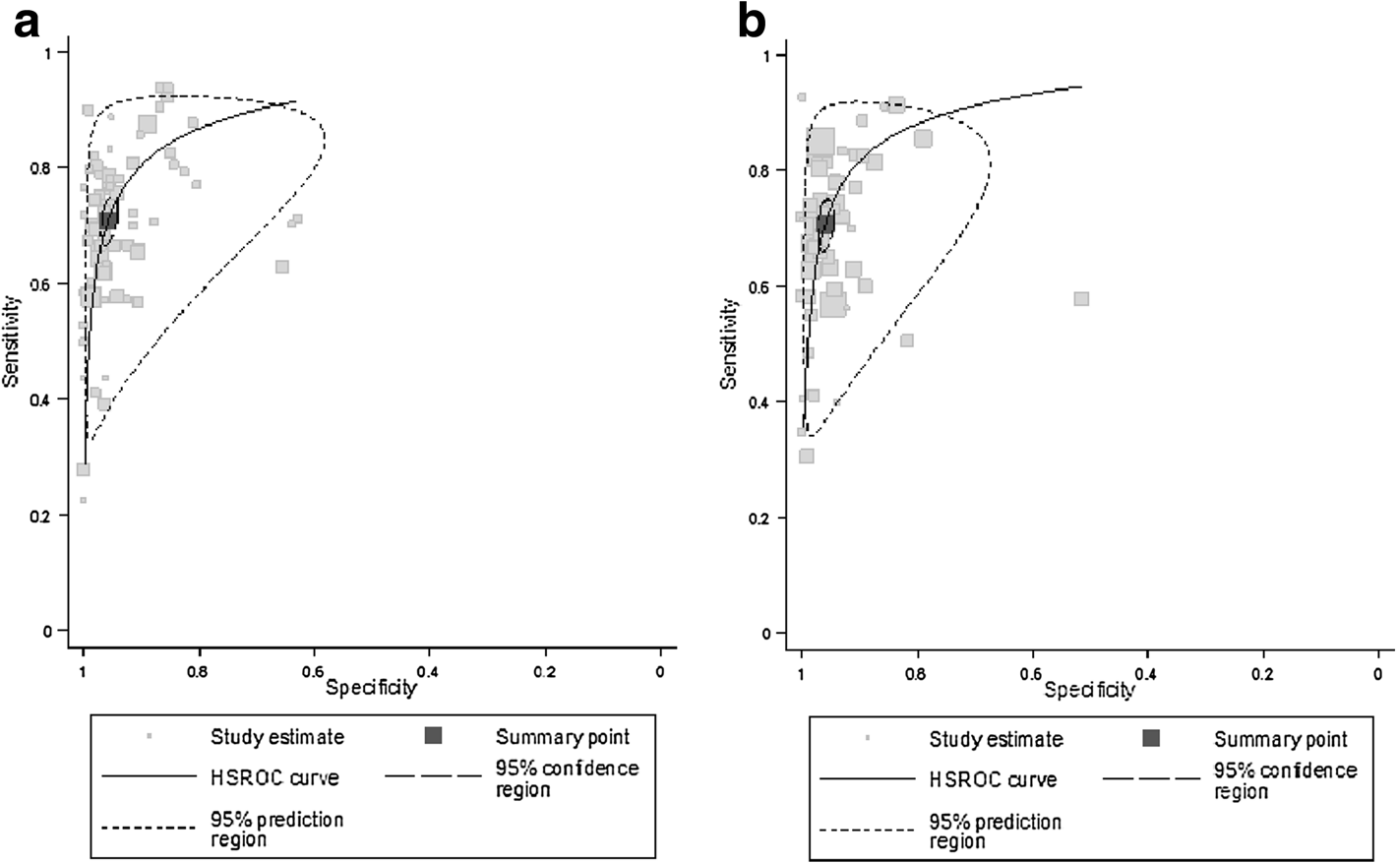
Hierarchical summary receiver-operating characteristic (HSROC) plots for a) high quality studies and b) low quality studies.

Overall, the pooled sensitivity and specificity were 70.8% (95% ci, 68.3% to 73.2%) and 95.8% (95% ci, 94.9% to 96.6%), respectively. Studies with high quality score produced similar results with studies with low quality score [sensitivity: 70.9% (95% ci, 67.5% to 74.1%) vs. 70.7% (95% ci, 67.0% to 74.1%) and specificity: 95.7% (94.4% to 96.7%) vs. 96.0% (94.8% to 97.0%)]. In HSROC analysis, sensitivity and specificity in high vs. low quality studies were found approximately equal; though, a slightly reduced variance of logit-transformed sensitivity and specificity was found in the low quality studies (0.39 and 0.93, respectively) (Figures 2a and 2b).

In comparing specific items (“yes” vs. “not”), the estimates of pooled sensitivities were statistically significant for items 10 and 13 [p = 0.03 and p = 0.06 (marginal), respectively]. In addition, the estimates of pooled specificities were statistically significant for items 13 and 16 (p = 0.01 and p = 0.01, respectively).

## Discussion

The present study investigated the quality of reporting of studies using the anti-CCP2 assay in RA patients according to the STARD statement. The differences between high and low quality studies were explored. The effect of reporting quality on pooled estimates of diagnostic metrics was also examined. Our analysis focused on the reporting of methodological items (items in method and results’ sections). In total, the 103 articles (corresponding to 132 studies) covered a publication period of 23 years. Almost the articles used in our analysis were published after the introduction of STARD statement (only 4 of them were published during 2003, year of STARD appearance).

Although the overall reporting quality was relatively good (13 items were reported by 70% or more of the studies) there are some essential methodological aspects of the studies (such as number/training/expertise of persons executing the tests, readers’ blinding to results, information on recruitment, adverse events from performing the tests, handling of missing responses and outliers) that are seldom reported making it difficult for the reader to assess explicitly the validity of a study. Comparing the quality of reporting in high versus low quality articles, significant differences were seen in a relatively large number of methodological items (11 items referred to: study population, data collection, reference standard, definition of units/cut-offs, number/training/expertise of persons executing and reading the tests, methods for calculating diagnostic measures, dates of recruitment, clinical/demographic characteristics, information on recruitment, time interval between tests, estimates of diagnostic accuracy).

Overall, the STARD quality score (high/low) has no effect on pooled sensitivity and pooled specificity. However, the meta-analysis showed an effect for specific STARD items. Studies not reporting sufficiently the methods used in calculating the measures of diagnostic accuracy (item 1), may have overestimated the sensitivity. In addition, the reporting of demographic and clinical characteristics/features of the study population (items 13 and 16) has affected the effect size of specificity, i.e. they have overestimated it, indicating also a spectrum bias [19].

However, the findings of the present synthesis (sensitivity of anti-CCP2, 71% and specificity, 96%) are compatible with those of earlier reviews (Nishimura et al. [27]: sensitivity, 67% and specificity, 95%, Whiting et al. [13]: sensitivity, 67%, specificity, 96%). An overestimation of our overall sensitivity might be resulted because of the lack of stratification by study design or disease duration in the analysis.

In a recent review, Whiting et al. [13] compared the accuracy of ACPA with that of RF in diagnosing RA in patients with early symptoms of the disease. They also assessed their studies for methodological quality by using a modification of the QUADAS criteria (items related to reporting quality, were removed). However, the impact of quality effect in diagnostic accuracy was not evaluated further. Nevertheless, the primary aim of the present study was to evaluate the effect of quality of reporting (according to STARD) in diagnostic accuracy rather than evaluating the effect of methodological quality (according to QUADAS); though, both tools can be useful for assessing the quality of diagnostic studies in a different perspective [28].

Applications of the STARD statement guidelines for assessing the quality of reporting in diagnostic accuracy studies, have been conducted in various medical fields such as in the field of diagnostic endoscopy [29], of juvenile idiopathic arthritis in peripheral joints [30], of diabetic retinopathy screening [31], of glucose monitor studies [32], of optical coherence tomography in glaucoma [33], of ultrasonography for the diagnosis of developmental dysplasia of the hip [34] and in the field of screening ultrasonography for trauma [35].

A limitation of the present study is that the literature search was restricted to PubMed. In addition, some studies may have been missed since we included only studies that provided data to estimate both sensitivity and specificity. However, the number of articles used is relatively large and an overview of reporting quality of studies may be obtained and the reached conclusions are unlikely to be affected by omitted studies. We would like to stress that lack of reporting of a STARD item does not necessarily implies that this item was not performed. Thus, a badly performed but well reported study will necessarily receive full credit. Finally, the published studies have had different design settings, and involved different stages of rheumatoid arthritis (study design, disease duration) which may question the synthesis of information, and therefore, the generalizability of results.

In conclusion, our attempt to assess the reporting quality of diagnostic accuracy studies in RA highlights the need for further improvement. Implementation of the quality reporting statements (e.g. CONSORT) have already improved the quality of reporting in other fields of medical research [36]. Thus, guidelines on the reporting of diagnostic accuracy studies are expected to improve the quality of reports of diagnostic studies as well. Finally, the study quality has no effect on the pooled estimates of diagnostic accuracy.

## Competing interests

The authors declare that they have no competing interests.

## Authors’ contributions

EZ conceived and supervised the study; EZ and AP drafted the manuscript; AP extracted the data, EZ and AP performed the statistical analysis; DZ and MV extracted the data and performed the statistical analysis of the revised manuscript; EZ and MV drafted the revised manuscript. All authors read and approved the final manuscript.

## Pre-publication history

The pre-publication history for this paper can be accessed here:

http://www.biomedcentral.com/1471-2474/13/113/prepub

## Supplementary Material

Additional file 1 Table S1 Characteristics of the studies. Click here for file

Additional file 2 Table S2 Endorsement of STARD statement by journals. Click here for file
