# Supplementary material for: The reporting quality of studies investigating the diagnostic accuracy of anti-CCP antibody in rheumatoid arthritis and its impact on diagnostic estimates
Source: BMC Musculoskelet Disord. 2012 Jun 25;13:113. doi: 10.1186/1471-2474-13-113 (PMC3488511; doi:10.1186/1471-2474-13-113)
Supplement: Additional file 1 Table S1 — Characteristics of the studies. [file 1471-2474-13-113-S1.doc]

**Supplementary Table 1.** Characteristics of the studies

| **A/A** | **Study, Year (Reference)** | **Population** | **Setting** | **Assay Manufacturer** | **Mean or Median, std or**  **(min-max) Age of patients with RA, y/n** | **Women, with RA %** | **Controls** | **Cutoff value** | **Sensitivity, %** | **Specificity, %** |
| --- | --- | --- | --- | --- | --- | --- | --- | --- | --- | --- |
| 1 | Quinn, 2006 | England | Rheumatology clinic | Axis-Shield | 58.0±13.7/182 | 64.3 | Other rheumatic diseases (n=116) | NA | 80.8 | 91.4 |
| 2 | Fernández-Suárez, 2005 | Spain | Primary care | 1. Euro-Diagnostica  2. Inova  3. Axis-Shield | (56.1± 2.2)/53 | 75.5 | Other rheumatic diseases (n=25), healthy persons (n=50) | 1. 25 UI/mL  2. 20 UI/mL  3. 5 UI/mL | 1. 52.8  2. 58.5  3. 52.8 | 1. 100.0 2. 100.0 3. 100.0 |
| 3 | Kwok, 2005 | Korea | Rheumatology clinic | Inova | 56(22-86)/129 | 86.8 | Other rheumatic diseases (n=68), healthy persons (n=60) | 20 AU | 55.0 | 98.4 |
| 4 | Greiner, 2005 | Germany | Teaching hospital | 1. Inova  2. Euroimmun 3. Euro-  Diagnostica | 58.6(19-84)/87 | NA | Other rheumatic diseases (n=233) | 1. 20 AU  2. 5 AU  3. 25 AU | 1. 79.3  2. 80.5  3. 80.5 | 1. 97.0  2. 97.9  3. 97.9 |
| 5 | Sauerland, 2005 | Germany | Teaching hospital | Euroimmun | NA/231 | NA | Other rheumatic diseases (n=469) | 5 AU | 74.0 | 94.5 |
| 6 | Aotsuka_2005 | Japan | Teaching hospital | Axis-Shield | NA/131 | NA | Other rheumatic diseases (n=90) | 5.6 U/mL | 87.8 | 81.1 |
| 7 | Choi, 2005 | Korea | Rheumatology clinic | Tosho | 51(22-83)/324 | 84.9 | Other rheumatic diseases (n=251), healthy persons (n=286) | 3.8 U/mL | 72.8 | 95.7 |
| 8 | Garcia-Berrocal, 2005 | Spain | Teaching hospital | 1. Axis-Shield  2. Inova  3. Euro-  Diagnostica | 62.9±12.9/87 | 75.9 | Other diseases (n=46) | 1. 5 U/mL  2. 20 U/mL  3. 25 U/mL | 1. 71.3  2. 77.0  3. 79.3 | 1. 63.0  2. 80.4  3. 82.6 |
| 9 | Nell, 2005 | Austria | Cohort study | Axis-Shield | 50(18-83)/102 | NA | Undifferentiated arthritis (n=98) | 5 AU | 41.2 | 98.0 |
| 10 | Raza, 2005 | England | Rheumatology clinic | Axis-Shield | NA/42 | NA | Hyperlipidemia (n=20), other rheumatic diseases (n=62) | 10 UI/mL | 57.1 | 96.3 |
| 11 | De Rycke, 2004 | Belgium | Rheumatology clinic | Euro-Diagnostica | 63.5(30-84)/118 | 65.3 | Other rheumatic diseases (n=146) | 25 U/mL | 75.4 | 97.3 |
| 12 | Girelli, 2004 | Italy | Rheumatology clinic | Axis-Shield | 70.0/35 | 77.1 | HCV infection (n=14), other rheumatic diseases (n=28) | 5 U/ml | 71.4 | 95.2 |
| 13 | Grootenboer-Mignot, 2004 | France | Teaching hospital | Euro-Diagnostica | NA/265 | NA | Other rheumatic diseases (n=91) | 25 UI/mL | 63.0 | 91.2 |
| 14 | Hitchon, 2004 | Canada | Teaching hospital | Inter-Medico/Inova | NA/41 | NA | Undifferentiated arthritis (n=23) | 20 IU/ml | 63.4 | 65.2 |
| 15 | Lopez-Hoyos, 2004 | Spain | Teaching hospital | Euro-Diagnostica | 45.9±10.2/41 | 70.7 | Polymyalgia rheumatica (n=49) | 50 IU/ml | 92.7 | 100.0 |
| 16 | Bombardieri, 2004 | Italy | Teaching hospital | Axis-Shield | 60(35-75)/30 | 70.0 | HCV infection (n=39) | 5 U/mL | 76.7 | 100.0 |
| 17 | Nielen, 2005 | Netherlands | Rheumatology clinic | Euro-Diagnostica | 57.6±14.8/258 | 70.2 | Undifferentiated arthritis (n=121) | 25 AU/ml | 57.8 | 94.2 |
| 18 | Dubucquoi, 2004 | France | Teaching hospital | Axis-Shield | NA/140 | NA | Other rheumatic diseases (n=98), healthy persons (n=33) | Manufacturer’s instructions | 64.3 | 98.5 |
| 19 | Söderlin, 2004 | Sweden | Health care centers | Euro-Diagnostica | 58.0±13.0/16 | 62.5 | Undifferentiated arthritis (n=10),  other rheumatic diseases (n=43) | 25 U/mL | 43.8 | 96.2 |
| 20 | Vallbracht, 2004 | Germany | Teaching hospital | Euro-Diagnostica | 62.4±14.7/295 | 80.3 | Degenerative joint disease (n=163), other rheumatic diseases (n=103), healthy persons (n=154) | 25 AU | 64.4 | 97.1 |
| 21 | Vittecoq, 2004 | France | Cohort study | Euroimmun | NA/176 | NA | Undifferentiated  arthritis (n=75), other rheumatic  diseases (n=63) | 5AU | 39.2 | 96.4 |
| 22 | Lee, 2003 | United States | Teaching hospital | Axis-Shield | 55.4(24-86)/103 | 84.5 | Noninflammatory arthritis (n=23), other rheumatic diseases (n=123) | 5AU | 66.0 | 90.4 |
| 23 | Rantapáä-Dahlqvist, 2003 | Netherlands | Cohort study | Euro-Diagnostica | 53.5(27-68)/67 | NA | Healthy age- and sex-matched persons (n=382) | 25 U/ml | 70.1 | 98.2 |
| 24 | Suzuki, 2003 | Japan | Teaching hospital | Axis-Shield | 60(19-83) /549 | 82.0 | Other rheumatic diseases (n=208) | Manufacturer’s instructions | 87.6 | 88.9 |
| 25 | Aflaky, 2010 | Iran | Teaching hospital | Euro-Diagnostica | 49.7±12.7/139 | 82.7 | Other rheumatic diseases (n=131) | 49 U/ml | 82.7 | 90.8 |
| 26 | Alexiou, 2007 | Greece | Teaching hospital | Inova Diagnostics | 60.3±12.8/155 | 76.1 | Other rheumatic diseases (n=178), healthy persons (n=100) | 20 IU/ml | 63.2 | 95.0 |
| 27 | Coenen, 2007 | Belgium | Rheumatology clinic | 1. Euro-  Diagnostica  2. Euroimmun  3. Phadia | NA/102 | NA | Other diseases (n=196) | 1. 25 U/mL  2. 5 RU/mL  3. 7 Elia U/mL | 1. 76.5  2. 72.5  3. 77.5 | 1. 95.4  2. 96.4  3. 95.9 |
| 28 | van der Cruyssen, 2008 | Belgium | Teaching hospital | 1. Euro-  Diagnostica  2. Pharmacia | NA/86 | NA | Other diseases (n=450) | 1. 42 U/mL  2. 7 U/mL | 1. 62.8  2. 67.4 | 1. 98.5  2. 98.7 |
| 29 | Damjanovska, 2010 | Netherlands | Teaching hospital | Euro-Diagnostica | 56.9±15.0/566 | 67.0 | Other diseases (n=351), healthy persons (n=99) | 25 U/ml | 56.9 | 94.7 |
| 30 | Dejaco, 2006 | Austria | Teaching hospital | Phadia | 60.4±12.0/164 | 85.4 | Other rheumatic diseases (n=303) | 10 U/mL | 70.1 | 98.7 |
| 31 | Enriconi dos Anjos, 2009 | Brazil | Teaching hospital | Inova | 49.1±12.3/70 | 91.4 | Systemic lupus erythematosus SLE (n=34), healthy persons (n=54) | 20 U/mL | 78.6 | 94.3 |
| 32 | Inanc, 2007 | Turkey | Teaching hospitals | Euro-Diagnostica | 51.0±12.0/208 | 83.2 | Psoriatric arthritis PsA (n=56), healthy persons (n=39) | Manufacturer’s instructions | 57.7 | 51.6 |
| 33 | Korkmaz, 2006 | Turkey | Teaching hospital | Euroimmun | NA/154 | 77.9 | Healthy persons (n=64) | 5 RU/mL | 72.1 | 100.0 |
| 34 | Kudo-Tanaka, 2007 | Japan | Cohort study | Axis-Shield | 63(12-79)/18 | 72.0 | Other rheumatic  diseases (n=54),  undifferentiated  arthritis (n=60) | 4.6 U/mL | 83.3 | 93.0 |
| 35 | Lutteri, 2007 | Belgium | Teaching hospital | 1. Inova  2. Euroimmun  3. Axis-Shield  4. Euro-  Diagnostica  5. Edia Euro-  Diagnostica  6. Pharmacia | 56(20-79)/120 | 71 | Other rheumatic  diseases (n=170) | 1. 20 U/mL  2. 5 U/mL  3. 5 U/mL  4. 25 U/mL  5. 5 U/mL  6. 10 EU/mL | 1. 66.7  2. 66.7  3. 66.7  4. 66.7  5. 66.7  6. 68.5 | 1. 94.4  2. 97.7  3. 97.7  4. 92.2  5. 97.0  6. 96.5 |
| 36 | Mori, 2009 | Japan | Teaching hospital | Axis-Shield | 57.5±13.3/106 | 78.3 | Active tuberculosis (n=89), healthy persons (n=237) | 5 U/mL | 82.1 | 97.9 |
| 37 | Abdel-Nasser, 2008 | Egypt | Teaching hospital | Inova Diagnostics | 42.5±13.1/50 | 88.0 | Other rheumatic  diseases (n=37),  healthy persons (n=10) | 30 U/mL | 70.0 | 91.5 |
| 38 | Ribeiro, 2008 | Brazil | NA | Euro- Diagnostica | 45.3±13.0/69 | 89.9 | Leprosy patients (n=158), healthy persons (n=89) | 25 U/mL | 81.2 | 97.2 |
| 39 | Santiago, 2008 | Canada | Health care centers | Inova Diagnostics | NA/48 | NA | Primary biliary cirrhosis PBC (n=80), systemic sclerosis SSc (n=74), healthy persons (n=40) | 20 U/mL | 77.1 | 90.7 |
| 40 | Sghiri, 2007 | Tunisia | Teaching hospital | Euroimmun | 48.2(17-78)/164 | 81.1 | Other rheumatic  diseases (n=152),  other diseases (n=99), healthy persons (n=92) | 5.25RU/mL | 78.7 | 95.6 |
| 41 | Sghiri, 2008 | Tunisia | Teaching hospital | Euroimmun | 45(17-78)/170 | 81.8 | Other rheumatic  diseases (n=158),  other diseases (n=92), healthy persons (n=59) | NA | 72.4 | 96.1 |
| 42 | Silveira, 2007 | Brazil | Teaching hospital | Inova Diagnostics | 56.0±15.0/132 | 78.8 | Other rheumatic  diseases (n=404),  other diseases (n=232) | 20 IU/mL | 62.1 | 96.5 |
| 43 | Vanichapuntu, 2010 | Thailand | Teaching hospital | Euro- Diagnostica | 49.5±13.9/125 | 92.8 | Other rheumatic  diseases (n=60),  healthy persons (n=60) | 25 U/mL | 58.4 | 100.0 |
| 44 | Wagner, 2009 | Austria | Rheumatology clinics | Abbott Laboratories Inc | 65.3(36-89)/193 | 80.8 | Other rheumatic  diseases (n=332) | 5 U/mL | 69.4 | 97.6 |
| 45 | Yamane, 2008 | Japan | Cohort study | Axis-Shield | NA/209 | 69.9 | Other rheumatic  diseases (n=52), other diseases (n=76), healthy persons (n=98) | 5 U/mL | 65.6 | 90.7 |
| 46 | Soós, 2007 | Hungary | Rheumatology clinic | Inova Diagnostics | 52.7±12.5/119 | 84.0 | Other rheumatic  diseases (n=74),  healthy persons (n=44) | 20 U/mL | 66.4 | 98.3 |
| 47 | Hill, 2006 | England/Canada | Rheumatology clinic | Inova Diagnostics | 58(32-87)/65 | 77.0 | Other rheumatic  diseases (n=112) | 25 RU/mL | 81.5 | 95.5 |
| 48 | Liu, 2009 | China | Teaching hospital | Fuchun-Zhongnan Biotech Co. | 53(16-83)/170 | 70.0 | Other rheumatic  diseases (n=66), other diseases (n=10), healthy persons (n=60) | 25 U/mL | 61.8 | 96.3 |
| 49 | Mutlu, 2009 | Turkey | Teaching hospitals | 1. Inova  Diagnostics  2. Axis-Shield | 53.8(26-76)/93 | 83.3 | Other rheumatic  diseases (n=66),  healthy persons (n=17) | 1. 20 U  2. 5 U/mL | 1. 60.2  2. 80.6 | 1. 98.8  2. 84.3 |
| 50 | Wu, 2007 | United States | Rheumatology clinic | Inova Diagnostics | NA/115 | NA | Other rheumatic  diseases (n=398), healthy persons (n=51) | NA | 70.4 | 97.8 |
| 51 | Snijders, 2008 | Netherlands | Rheumatology clinic | Euro- Diagnostica | 62.8±13.4/109 | 74.3 | Other rheumatic  diseases (n=351),  healthy persons (n=420) | 25 U/mL | 57.8 | 98.7 |
| 52 | Lopez_Longo, 2006 | Spain | Teaching hospital | Euro- Diagnostica | NA/87 | NA | Other rheumatic  diseases (n=163) | 25 U/mL | 72.4 | 94.5 |
| 53 | Nikolaisen, 2007 | Norway | Teaching hospital | Inova Diagnostics | 61/86 | NA | Oher rheumatic diseases (n=148) | 20 AU/mL | 65.1 | 95.3 |
| 54 | Wild, 2008 | mainly UK | Clinical centers | Axis-Shield | NA/184 | NA | Other rheumatic diseases (n=274) | 5 U/mL | 75.8 | 94.0 |
| 55 | Sène, 2006 | France | Teaching hospital | Euro- Diagnostica | 58.6±14.1/64 | 87.5 | HCV infection (n=147) | 25 U/mL | 78.1 | 94.0 |
| 56 | Amezcua-Guerra, 2006 | Mexico | Rheumatology clinic | Inova Diagnostics | NA/14 | 50.0 | Systemic lupus erythematosus (SLE) (n=7) , healthy persons (n=7) | 60 U/mL | 71.4 | 100.0 |
| 57 | Ates, 2007 | Turkey | Rheumatology clinic | Euroimmun | NA/32 | 90.6 | Other rheumatic diseases (n=30) | 5 RU/mL | 40.6 | 100.0 |
| 58 | Atta, 2007 | Brazil | NA | Inova Diagnostics | NA/10 | NA | Visceral leishmaniasis (VL) (n=10), healthy persons (n=40) | 20 IU | 40.0 | 94.0 |
| 59 | Bassyouni, 2009 | Egypt | Teaching hospital | Euroimmun | 39.2±10.6/30 | NA | Chronic hepatitis C virus (HCV) infection (n=47) | 5 RU | 70.0 | 91.5 |
| 60 | Bizzaro, 2007 | Italy | Teaching hospitals | 1. Aesku  Diagnostika  2. Axis-Shield  3. Euro-  Diagnostica  4. Euroimmun  5. Orgentec  6. Phadia  7. Inova  Diagnostics | 64(40-86)/100 | 90.0 | Other rheumatic diseases (n=51), infectious diseases (n=39), other diseases (n=38)  healthy persons (n=74) | 1. 68 AU  2. 5 AU  3. 25 AU  4. 5 AU  5. 20 AU  6. 20 AU  7. 20 AU | 1. 60.0  2. 71.0  3. 75.0  4. 73.0  5. 72.0  6. 74.0  7. 66.0 | 1. 89.1  2. 97.0  3. 97.0  4. 98.5  5. 93.1  6. 98.5  7. 97.0 |
| 61 | Candia, 2006 | Colombia | Rheumatology clinic | Inova  Diagnostics | NA/41 | NA | Cutaneous psoriasis (n=106), other rheumatic diseases (n=156), healthy persons (n=41) | 20 UΙ/mL | 78.0 | 94.1 |
| 62 | Ceccato, 2006 | Argentina | Rheumatology clinic | Inova  Diagnostics | NA/16 | 75.0 | Polymyalgia rheumatica (PMR) (n=13) | 20 U/mL | 56.3 | 92.3 |
| 63 | Chibnik, 2009 | United States | Cohort study | Axis-Shield | 54.6±8.2/93 | 100.0 | Healthy persons (n=279) | 5 U/mL | 28.0 | 100.0 |
| 64 | Correia, 2008 | Portugal | NA | 1. Inova  Diagnostics  2. Phadia | NA/86 | NA | Autoimmune diseases (n=34), infections (n=30), other rheumatic diseases (n=26), | 1. 20 U/mL  2. 10 U/mL | 1. 73.3  2. 67.4 | 1. 94.4  2. 97.8 |
| 65 | de Seny, 2005 | Belgium | Teaching hospital | Euroimmun | 52(26-79)/34 | 64.7 | Other diseases (n=53), healthy persons (n=16) | 5RU/mL | 82.4 | 98.6 |
| 66 | Eshed, 2009 | Germany | Rheumatology clinic | Euro-  Diagnostica | 48(17-65)/58 | 25.9 | Other rheumatic diseases (n=41) | 25 IU | 70.7 | 87.8 |
| 67 | Ezzat, 2009 | Egypt | Rheumatology clinic | Inova  Diagnostics | 45.8±9.6/30 | 83.3 | HCV-related polyarthropathy (n=22) | 20 U/mL | 83.3 | 95.5 |
| 68 | Fusconi, 2005 | Italy | NA | Axis-Shield | 66(20-77)/89 | 74.2 | Liver-diseases (n=272) | 5AU | 59.6 | 94.5 |
| 69 | Gupta, 2009 | India | Rheumatology clinic | Euroimmun | 42.1±12.9/63 | 85.7 | Other rheumatic diseases (n=51) | 5AU | 85.7 | 90.2 |
| 70 | Havemose-Poulsen, 2006 | Denmark | Teaching hospital | Euro-  Diagnostica | 30.0±4.0/23 | 95.7 | Aggressive periodontitis (n=45), other rheumatic diseases (n=10), healthy persons (n=25) | 25AU/mL | 34.8 | 100.0 |
| 71 | Heidari B, 2009 | Iran | Rheumatology clinics | Euroimmun | 51.0±14.0/201 | 80.0 | Other rheumatic diseases (n=94), healthy persons (n=114) | 14.8 U/mL | 81.6 | 87.5 |
| 72 | Herold, 2005 | Austria | Teaching hospital | 1) Inova  Diagnostics  2) Pharmacia  Diagnostics | NA/66 | NA | Other rheumatic diseases (n=328) | 1. 20 U/mL  2. 7 U/mL | 1. 68.2  2. 80.3 | 1. 96.6  2. 97.0 |
| 73 | Hodkinson, 2010 | South Africa | Teaching hospitals | Phadia | 47.6±12.5/120 | 83.3 | SLE (n=35), SSc (n=28), healthy persons (n=30) | 10 U/mL | 82.5 | 84.9 |
| 74 | Hwang, 2010 | Korea | Teaching hospital | 1) Abbott  Diagnostics  2) Phadia | 59(25-77)/69 | 85.5 | Other diseases (n=40), viral infections (n=31), other rheumatic diseases (n=15) | 1. 5 U/mL  2. 10 Elia U/mL | 1. 76.8  2. 78.3 | 1. 95.3  2. 95.3 |
| 75 | Innala, 2008 | Sweden | Teaching hospital | Axis-Shield | NA/192 | NA | Healthy persons (n=102) | 5 U/mL | 74.5 | 98.0 |
| 76 | Ioan-Facsinay, 2008 | Canada | Rheumatology clinic | Euro-  Diagnostica | 52(19-76)/81 | 90.1 | UA (n=8), healthy persons (n=286) | NA | 91.4 | 83.7 |
| 77 | Jorgensen, 2008 | Norway | Blood donors from cohort study | Euro-  Diagnostica | 41(21-64)/49 | 63.3 | Healthy persons (n=245) | 25 AU/mL | 30.6 | 99.2 |
| 78 | Kastbom, 2004 | Sweden | Rheumatology centers | Euro-  Diagnostica | NA/235 | 68.6 | Other rheumatic diseases (n=7), healthy persons (n=80) | 25 U/mL | 66.8 | 97.7 |
| 79 | Kim, 2010 | Korea | Teaching hospital | 1. Axis-Shield  2. Abbott  3. Abbott  4. Roche  Diagnostics | NA/64 | 81.3 | Other diseases (n=152) | 1. 5 U/mL  2. 5 U/mL  3. 5 U/mL  4. 17 U/mL | 1. 93.8  2. 92.2  3. 93.8  4. 90.6 | 1. 85.5  2. 85.5  3. 86.8  4. 86.8 |
| 80 | Koivula, 2007 | Finland | Blood donors from cohort study | Euro-  Diagnostica | 45.6±12.3/89 | 68.5 | Other diseases (n=261) | 25 U/mL | 62.9 | 65.5 |
| 81 | Koivula, 2008 | Finland | Teaching hospital | Euro-  Diagnostica | 60.0±13.0/18 | 61.1 | UA (n=98), other rheumatic diseases (n=30) | 25 U/mL | 72.2 | 91.4 |
| 82 | Lim, 2009 | Korea | Teaching hospital | Axis-Shield | NA/10 | NA | Other diseases (n=230) | 5 U/mL | 90.0 | 99.1 |
| 83 | Limaye, 2005 | Australia | Teaching hospital | Inova  Diagnostics | NA/29 | NA | Other rheumatic diseases (n=5) | Manufacturer’s instructions | 86.2 | 80.0 |
| 84 | Liu, 2008 | China | Rheumatology clinic | Axis-Shield | 50.0±14.9/44 | 77.3 | HCV-infections (n=34), other rheumatic diseases (n=42) | 5 U/mL | 84.1 | 96.1 |
| 85 | Narvaez, 2008 | Spain | Teaching hospital | Euro-  Diagnostica or Phadia | 53.0±6.4/31 | 71.0 | UA (n=7), other diseases (n=2) | NA | 22.6 | 100.0 |
| 86 | Nell-Duxneuner, 2010 | Austria | Cohort study | Axis-Shield | NA/102 | 73.0 | UA (n=37), other diseases (n=61) | 5 AU | 41.0 | 98.0 |
| 87 | Panchagnula, 2006 | India | Rheumatology center | Euroimmun | NA/41 | NA | Other rheumatic diseases (n=102) | 5 U/mL | 63.4 | 96.1 |
| 88 | Qing, 2009 | China | Teaching hospital | Euroimmun | NA/76 | 76.3 | SLE (n=267), healthy persons (n=87) | 5 AU | 85.5 | 79.1 |
| 89 | Rodriguez-Mahou, 2006 | Spain | Teaching hospital | Euro-  Diagnostica | NA/56 | 23.2 | Other rheumatic diseases (n=28) | 25 U/mL | 91.1 | 85.7 |
| 90 | Ryu, 2009 | Korea | Teaching hospital | Axis-Shield | NA/100 | NA | Other rheumatic diseases (n=97), chronic viral hepatitis (n=200), healthy persons (n=834) | 5 U/mL | 85.0 | 96.7 |
| 91 | Salvador, 2003 | Spain | Rheumatology clinic | Euro-  Diagnostica | NA/81 | 74.1 | Other rheumatic diseases (n=122) | 50 U | 50.6 | 81.8 |
| 92 | Samanci, 2005 | Turkey | Rheumatology clinics | Euroimmun | 53.1±10.8/76 | 88.2 | Other rheumatic diseases (n=51), healthy persons (n=32) | 30 RU/mL | 48.7 | 98.8 |
| 93 | Shibata, 2009 | Japan | Teaching hospital | Axis-Shield | 61.1±9.1/9 | 100.0 | PsA (n=16), psoriasis (n=15), healthy persons (n=11) | 5 U/mL | 88.9 | 95.2 |
| 94 | Shidara, 2010 | Japan | Rheumatology clinic | Axis-Shield / Medical and Biological laboratories | 59.3±13.4/106 | 79.2 | Other rheumatic diseases (n=57) | 4.5 U/mL | 88.7 | 89.5 |
| 95 | Singh, 2010 | India | Teaching hospital | Aesku Diagnostics | NA/119 | 85.7 | Healthy persons (n=26) | 15 U/mL | 50.0 | 100.0 |
| 96 | Singwe-Ngandeu, 2010 | Cameroon | Rheumatology clinic | Euro-  Diagnostica | 53.5(39.0-61.5)/56 | 95.0 | Other rheumatic diseases (n=51), healthy persons (n=50) | 32 U/mL | 82.1 | 98.0 |
| 97 | Takasaki, 2004 | Japan | Teaching hospital | Axis-Shield | NA/104 | NA | Other rheumatic diseases (n=181) | 4.5 U/mL | 82.7 | 89.5 |
| 98 | Tamai, 2009 | Japan | Rheumatology clinic | Axis-Shield | 53(25-80)/75 | 77.3 | UA (n=54) | 4.5 U/mL | 57.3 | 92.6 |
| 99 | Tampoia, 2005 | Italy | Rheumatology clinic | Axis-Shield | 45(20-68)/83 | 80.7 | Other diseases (n=72), other rheumatic diseases (n=48), healthy persons (n=20) | 5 U/mL | 67.5 | 99.3 |
| 100 | Tanaka, 2010 | Japan | Local hospitals | Axis-Shield | NA/99 | NA | OA (n=16), healthy persons (n=94) | 4.5 U/mL | 79.8 | 99.1 |
| 101 | Aigner, 2007 | Austria | Rheumatology clinic | Euroimmun | 46.5±11.3/31 | 74.2 | Other diseases (n=87), healthy persons (n=162) | 5 RU/mL | 58.1 | 98.8 |
| 102 | Awwad, 2010 | Syria | Teaching hospitals | 1. Euroimmun 2. Genesis  3. Genesis | NA/64 | 71.9 | Healthy persons (n=25) | 1. 1 U/mL  2. 22 U/mL  3. 1 U/mL | 1. 71.9  2. 70.3  3. 43.8 | 1. 100.0  2. 64.0  3. 100.0 |
| 103 | Predeteanu, 2009 | Romania | Rheumatology clinic | NA | 54.6±12.3/111 | 87.4 | Other rheumatic diseases (n=106) | 20 U/mL | 56.8 | 90.6 |
