# Supplementary material for: The reporting quality of studies investigating the diagnostic accuracy of anti-CCP antibody in rheumatoid arthritis and its impact on diagnostic estimates
Source: BMC Musculoskelet Disord. 2012 Jun 25;13:113. doi: 10.1186/1471-2474-13-113 (PMC3488511; doi:10.1186/1471-2474-13-113)
Supplement: Additional file 2 Table S2 — Endorsement of STARD statement by journals. [file 1471-2474-13-113-S2.doc]

| **A/A** | **Journal** | **STARD**  **endorsement** | **Published articles**  **(n=103)** |  |
| --- | --- | --- | --- | --- |
| 1 | Ann Rheum Dis | Yes | 14 | Journals that endorse STARD statement  16/35 (45.7%)  Articles published in journals that endorse STARD statement  61/103 (59.2%) |
| 2 | Clin Rheumatol | Yes | 10 |
| 3 | Rheumatol Int | Yes | 9 |
| 4 | Rheumatology (Oxford) | No | 6 |
| 5 | Arthritis Research & Therapy | Yes | 4 |
| 6 | Arthritis & Rheumatism | Yes | 4 |
| 7 | Clin Chem Lab Med | Yes | 4 |
| 8 | Clinical Chemistry | Yes | 1 |
| 9 | Clin Rev Allergy Immunol | Yes | 2 |
| 10 | BMC Musculoskeletal Disorders | Yes | 1 |
| 11 | Semin Arthritis Rheumatol | Yes | 1 |
| 12 | Aliment Pharmacol Ther | Yes | 1 |
| 13 | Indian J Med Sci | Yes | 1 |
| 14 | J Korean Med Sci | Yes | 1 |
| 15 | J Periodontol | Yes | 1 |
| 16 | Clin Biochemistry | Yes | 1 |
| 17 | J Rheumatol | No | 12 | Journals that do not endorse STARD statement  19/35 (54.3%)  Articles published in journals that do not endorse STARD statement  42/103 (40.8%) |
| 18 | Scand J Rheumatol | No | 7 |
| 19 | J Clin Rheumatol | No | 3 |
| 20 | Ann N Y Acad Sci | No | 2 |
| 21 | Clin Chimica Acta | No | 4 |
| 22 | Clin Exp Rheumatol | No | 1 |
| 23 | Biomarkers | No | 1 |
| 24 | Eur Ann Allergy Clin Immunol | No | 1 |
| 25 | Clin Exp Immunol | No | 1 |
| 26 | J Autoimmun | No | 1 |
| 27 | Clin Dev Immunol | No | 1 |
| 28 | Brazil J Med Biol Res | No | 1 |
| 29 | Lupus | No | 1 |
| 30 | J Natl Med Assoc | No | 1 |
| 31 | J Dermatol Sci | No | 1 |
| 32 | Mod Rheumatol | No | 1 |
| 33 | Clin Lab | No | 1 |
| 34 | Ann Saudi Med | No | 1 |
| 35 | J Med Life | No | 1 |

**Supplementary Table 2.** Endorsement of STARD statement by journals
